# Reasoning on Figurative Language: A Preliminary Study on Children with Autism Spectrum Disorder and Klinefelter Syndrome

**DOI:** 10.3390/brainsci9030058

**Published:** 2019-03-11

**Authors:** Sergio Melogno, Maria Antonietta Pinto, Teresa Gloria Scalisi, Margherita Orsolini, Luigi Tarani, Gloria Di Filippo

**Affiliations:** 1 Department of Psychology of Development and Socialization Processes, “Sapienza”, University of Rome, 00185 Rome, Italy; mariantonietta.pinto@uniroma1.it (M.A.P.); gloria.scalisi@uniroma1.it (T.G.S.); margherita.orsolini@uniroma1.it (M.O.); 2 Department of Pediatrics, “Sapienza”, University of Rome, 00185 Rome, Italy; luigi.tarani@uniroma1.it; 3 Faculty of Psychology, University “Niccolò Cusano”, Telematica, 00166 Rome, Italy; gloria.difilippo@unicusano.it

**Keywords:** idioms, metaphors, comprehension, autism spectrum disorder, Klinefelter syndrome, children

## Abstract

In this study we explored metaphor and idiom competencies in two clinical populations, children with autistic spectrum disorder (ASD) and children with Klinefelter syndrome (KS), (age range: 9–12), compared to typically developing (TD) children of the same age. These three groups were tested with two multiple-choice tests assessing idiom comprehension through iconic and verbal alternatives and a metaphor comprehension test composed of novel, physical-psychological metaphors, requesting verbal explanations. To these instruments, another test was added, assessing basic sentence comprehension. Performances on the different linguistic tasks were examined by means of discriminant analysis which showed that idiom comprehension had a very small weight in distinguishing children with ASD from TD controls, whereas metaphor explanation did distinguish them. This study suggests that figurative language comprehension is not a “core deficit” per se in individuals with ASD. Only when the task requires to explicitly construct and explain a semantic mapping between the two terms of a metaphor does the performance of children with ASD significantly deviate from the typical population. These results are interpreted in terms of a difficulty in children with ASD and KS with complex cognitive and linguistic processes and also in relation with clinical assessment.

## 1. Introduction

Figurative language is often used as an umbrella term to refer to all the expressions—from single words to complete sentences—whose interpretation requires to go beyond the literal meaning of every lexical constituent and retrieve the interlocutor’s communicative intention appropriately [1]. In this article, we will refer to two main categories: metaphors and idioms, which, in turn, comprise a plurality of varieties.

For what concerns metaphor, many types of single lexical items such as nouns, adjectives, and verbs may be used with a metaphorical meaning, and also be combined to form metaphorical sentences. A paradigmatic form of such sentences is the so-called ‘nominal metaphor’, which is formed by two components, A and B, where A is called the ‘tenor’ [2] and B is called the ‘vehicle’. For instance, when we say that “Intelligence is a skyscraper”, we mean that A (‘Intelligence’) can be viewed as something that can reach a noticeable height, floor after floor, which enables the individual who ascends them to catch a wider and more complete sight of things, just as a skyscraper (B, in this case) gives us the possibility to do. This sentence is also an example of ‘novel’ metaphor, i.e., a freshly created metaphor, not commonly used and therefore not yet lexicalized. The novelty of these expressions generates surprise because the respective meanings of the basic constituents, tenor and vehicle, are associated in such a way as to create a semantic conflict. However, in order to solve this conflict, the interpreter may analyze the meanings of tenor and vehicle and try to find a certain number of semantic features that can be shared by both, technically called ‘common ground’ [2]. In the above example, height, gradual elevation, widening of sight and horizons can be seen as features shared by both intelligence and skyscrapers. Novel metaphors can potentially become lexicalized, once their usage is conventionalized and becomes widespread.

Several authors argued that metaphors are language ‘devices’ that support the building of abstract concepts [3,4,5,6]. For instance, when faced with the nominal metaphor “Intelligence is a skyscraper”, children might grasp some abstract features of the word “intelligence” by applying their knowledge about the more concrete word “skyscraper”. Some authors view the semantic mapping between tenor and vehicle as grounded on analogical reasoning [7,8,9,10]. In this vein, metaphor comprehension would rely on semantic inferences to align tenor and vehicle. Like in problem-solving, inferences are necessary to construct a consistent relationship between entities distant from each other.

Unlike metaphors, and novel metaphors in particular, the figurative characteristics of idioms are always conventionalized, and can be felt by locutors as being more or less transparent. For example, the idiom “To lose oneself in a glass of water” can suggest by itself the incapability to face modest difficulties, contrary to other cases, such as, in English: to “spill the beans” = to reveal a secret, or “to kick the buck” = to die, or, in Italian “con questi chiari di luna!” (literally, in English: “with these moonlights”) = in such difficult times. Concerning idiom comprehension, the theoretical debate essentially opposes two positions, one that points to lexical access and/or retrieval, and the other that suggests that idioms are represented in a distributed way and processed as complex expressions. (see Swinney and Cutler [11], and Vulchanova et al. [12].)

### 1.1. Autism Spectrum Disorder and Klinefelter Syndrome

Impairments in interaction and social communication, and restricted, repetitive behavior and interests are distinctive characteristics of the autistic spectrum disorder (ASD) condition [13]. In this neurodevelopmental disorder, we can detect the presence of problems in interaction through deficits in the use of eye contact, facial expression and emotion reading, body posture, and gestures. The combination of these deficits are an obstacle to the development of social and emotional reciprocity. Individuals with ASD characterized by fluent language show difficulties in conversation, and stereotyped and repetitive language usages. In association with repetitive and stereotyped interests we often find general rigidity and non-functional routines and rituals. Other distinctive behavioral patterns are motor mannerisms and hypo- and/or hyper-reactivity to sensory inputs.

Actually, many atypical cognitive profiles [14] can be found in the ASD condition, where linguistic competencies and other cognitive capabilities can range from very poor to acceptable, in spite of some evident difficulties in pragmatic usages, as in figurative language.

KS, the most frequent gonosomic anomaly, is a testicular dysgenesis due to a gonosomic polysomy as 47,XXY (80%), or 48,XXXY, 48,XXYY, 49,XXXXY, or 46,XX/47,XXY (20%). The incidence is 1:600 male born. Klinefelter syndrome (KS) is frequently under-diagnosed due to its high clinical variability. In fact, the characteristic hypogonadic eunuchoid phenotype may be observed less frequently than an almost normal phenotype, characterized by the only constant clinical signs as microrchidia (small testicles: <10 mL in adulthood) and azoospermia (absence of spermatozoa in the seminal fluid), which can be detected in adulthood, especially in the case of infertility. During childhood, suspicion can be advanced in cases of cryptorchidism (a condition in which one or both testicles fail to move from the abdomen, where they develop before birth, down in the scrotum), hypogenitalism (subnormal development of genital organs), hypospadias (an abnormality of the penis in which the urethra opens on the undersurface), and the length of the lower compared to upper limbs. At puberty, evaluation often attests low volume of testes (<3 mL at 11 years), pubertal delay, modest development of secondary sexual characters, and the appearance of gynecomastia. Clinical diagnosis is then confirmed by the examination of the karyotype and the hormonal dosage (hypergonadotropic hypogonadism).

For what concerns the cognitive profiles of individuals with KS, a relevant aspect underlined by the majority of the researchers is a clear delay in basic language competencies, attention and executive deficits, learning difficulties, and, in some cases, autistic-like symptoms [15,16].

### 1.2. Understanding Metaphors and Idioms in Children with ASD and KS

Metaphor comprehension in individuals with ASD has been extensively studied from the early 1990s in a search for factors likely to be correlated to this particular type of semantic processing. These studies were partly based on experimental research [17,18,19,20,21,22,23], partly on case studies [24], reviews [12,25,26], and partly on meta-analyses [27].

The pioneering study by Happé [17] explored, for the first time, the relationship between metaphor comprehension and Theory of Mind (ToM). Eighteen individuals with ASD, aged 9 to 28 years, were subdivided into three subgroups based on their performance on first and second order false belief tasks. Results showed different performances when participants were requested to complete sentences by choosing between multiple alternatives in three experimental conditions: synonyms, similes, and metaphors. Success with first-order false belief task was found to be correlated with metaphor comprehension. These results were interpreted in light of the relevance of the ToM factor in figurative language comprehension.

Nevertheless, when Norbury [19] used the same tasks with some adaptations and tested semantic abilities in parallel, he found that ToM was a necessary but not sufficient condition for metaphor comprehension. In his study, a sample of 94 children (age range: 8–15) was subdivided into three subgroups on the basis of the presence/absence of ASD and linguistic impairments. The first group had language disorders without ASD, the second had ASD without language disorders and the third had both ASD and language disorders. The results showed lower performances only in the first and the third subgroups compared to controls. Further analyses also showed that semantic abilities had a stronger power to predict metaphor comprehension than ToM. However, this result was criticized by Rundblad and Annaz [20] who pointed out that the test used to measure semantic knowledge in Norbury’s research included a certain number of subtests assessing figurative language comprehension. In other words, what was tested in reality was figurative language comprehension.

In a more recent study, Kasirer and Mashal [28] administered the same type of tasks to 34 children with ASD and 39 TD children. The results were that the ASD group understood fewer conventional metaphors than the TD control group but no differences appeared in novel metaphors. A similar contrast between novel and conventional metaphors was found in the generation task in favor of the ASD group, who produced more creative metaphors than the TD peers. In their meta-analysis, Kalandadze et al. [27], emphasized the heterogeneity of the results available to date and suggested that deficits in figurative language are neither applicable to all individuals with ASD nor specific to this population. These heterogeneous results could be explained by two main factors. The first is methodological, and has to do with the assessment modalities adopted by the researcher, which can range from multiple-choice tasks with either verbal or iconic alternatives, to requests of verbal explanation. The second factor concerns the typologies of metaphors presented in the tasks, which can vary as a function of linguistic categories (for example, the above distinction between novel versus lexicalized metaphors), and the degree of abstraction of the tenor-vehicle relationship, as it occurs in sensory versus physical-psychological metaphors [29]. An example of the former is “The sun is an orange” [8], while an example of the latter is “The prison guard is a rock” [29]. Physical-psychological metaphors are cognitively more demanding than sensory metaphors, because the semantic gap between vehicle and tenor calls for explicit analogical reasoning, whereas sensory metaphors can be processed more intuitively, based on perceptual characteristics [30].

Concerning idiom comprehension, Kerbell and Grunwell [31] and Norbury [32] found that children with ASD experienced considerable difficulties compared to controls. Norbury, in particular, pointed to the relevance of linguistic factors, as the two subgroups of children with ASD, those with and those without linguistic impairments understood idioms at a significantly different level. Overall, Norbury’s study [32] showed that idiom comprehension can be predicted by syntax, vocabulary, ToM, and working memory. White et al. [33] showed that children with ASD, aged 5 to 12, had lower performances on idiom comprehension than TD controls. Nevertheless, when these participants with ASD were matched to younger TD children by the level of syntax comprehension, performances were very similar. In sum, idiom comprehension seems to be more correlated to the level of linguistic development in children with ASD than to their cognitive profile. This correlation could be due to the fact that idiom comprehension heavily relies on the retrieval of conventionalized meanings.

For what concerns the population with KS, according to Bishop and Sherif’s review [16], studies are essentially focused on basic linguistic competencies, both in comprehension and production. Deficits are more marked at expressive than at receptive level [34] and typically include weaknesses in word finding, expressive grammar and speech. Since these deficits also affect literacy skills they also negatively reverberate on academic achievement [35,36]. However, other researchers explored high-level linguistic competencies. Among these, we will cite Ross and colleagues [35], who studied figurative language abilities in a sample of 50 participants composed of children and adolescents (age range: 4.1–17.8) using the Wiig and Secords Test of Language Competence Expanded Edition (TLC-E) [37]. This test includes ambiguous sentences, listening comprehension, oral expression, and figurative language subtests. Significant differences were found between children under and above the age of 10, the former showing average performances in figurative language whereas the latter performed at a border level. In a similar vein, Melogno et al. [38] studied figurative language abilities in addition to other high-level language competencies and ToM in a small group with KS (age range: 9–13) compared to a TD control group. The major differences were on both ToM and a task that requested to explain physical-psychological metaphors. It is therefore possible that ToM and high-level linguistic competencies are two problematic areas for both children with KS and ASD. For example, Bishop et al. [39] reported that 9 out of 19 children prenatally diagnosed as KS followed a language therapy, and 2 of these children were diagnosed as autism spectrum disorder. Bruining et al. [40], in a sample of 12 Dutch children with KS, found that 65% fulfilled the criteria to be included in language disorders, 63% to be included in attention deficit disorder, and 27% in ASD. The presence of autistic-like symptoms in some individuals with KS induced researchers [41,42] to investigate on their ToM capabilities, such as inferring mental states and recognizing emotions. Individuals with KS seem less accurate in detecting socio-emotional cues such as facial expressions and recognizing emotions from prosody [41,42].

In this study, we analyzed idiom and metaphor competencies in children with ASD and KS compared to a TD control group. The comparison between the two clinical populations was justified by the fact that language development can be a critical area for both. Nevertheless, as we have seen above (1.2) there is a certain asymmetry in the studies exploring figurative language because children with ASD received far more attention than children with KS. Our contribution aims at enriching the picture about these two populations. To this end, we used two types of tasks, one relying on multiple choice and the other, on verbal explanation. We wondered to what extent the profiles of these two groups could overlap. We expected both groups of children, those with ASD and those with KS, to show levels of idiom comprehension, as assessed by a multiple-choice task, comparable to those of their TD peers. On the contrary, we expected the same children to perform lower than the TD controls on metaphor explanation, as assessed by a task that requests analogical reasoning based on explicit verbalization.

## 2. The Study

### 2.1. Participants

Three groups of 10 children each (age range: 9–12 years), all males, participated in this study. To verify if there were mean age differences between the three groups (expressed in months), a one-way ANOVA was run, that showed no significant difference (*F*_(2, 27)_ = 0.9; *p* = 0.417). The first group (mean age: 124.1; SD: 14.0) was composed of children with ASD, the second (mean age: 122.9; SD: 13.7) of children with KS, and the third, of TD controls (mean age: 130.2; SD: 11.2). The first and the second group had previously received their diagnosis and were recruited at the Department of Pediatrics and Child Neuropsychiatry; XXXX). Some of the children with ASD were diagnosed at preschool age on the basis of *Diagnostic and Statistical Manual of Mental Disorders*, IV (DSM IV) [43], and the diagnosis was reconfirmed on the basis of *Diagnostic and Statistical Manual of Mental Disorders* 5 (DSM 5) [13]. In both cases, the instruments used for the diagnosis included *Autism Diagnostic Observation Schedule 2* (ADOS 2) [44,45] and *Autism Diagnostic Interview-Revised* (ADI-R) [46,47]. Two criteria were used to include participants in the respective groups, namely age range (9–12) and total Intelligence Quotient (IQ) > 71, as measured by the Wechsler Intelligence Scale (WISC IV) [48,49].

All the children belonging to the ASD and KS group were enrolled in inclusive schools in the grades corresponding to their age, and were therefore exposed to the same type of everyday linguistic usage. Regarding the TD controls, as we were not allowed to administer the WISC IV [48], we obtained general information concerning development and learning through a questionnaire addressing teachers and parents (See Appendix A, Table A1), which revealed an absence of emotional or other developmental disorders, and adequate learning abilities. Their socio-cultural milieu was very similar to the one of the sample with KS and ASD, based on both parents’ educational level (at least one parent with post-secondary diploma). The total sample was composed of native Italian-speaking children.

### 2.2. Assessment Tools

We assessed figurative language competencies and basic sentence comprehension with the following tests, devised for Italian-speaking children:

(1) A Metaphor Comprehension Test (MCT) for children aged 9 to 14, the MCT (It: TCM) [50].

The MCT is composed of 12 metaphorical items in the ‘X is Y’ form. Ex: “Intelligence is a skyscraper”. Metaphor comprehension is viewed as the solution of a semantic conflict that underlies each metaphor, a process that can be implemented at different levels. The assessment scale comprises four of these levels, corresponding to the following scoring.

- Level 0 (score: 0): the semantic conflict is either denied or refused. Refusals and/or denials can take the following form. (a) No answer: blanks or “I don’t know” answers; (b) “No, it’s nonsense”; (c) Metonymic answers: “A skyscraper is full of people who work in it”; (d) Answers exclusively focused on vehicle or on tenor: “If you are intelligent you are grown up and tall”, “It (“intelligence”) never stops functioning”, “It (“the skyscraper”) can go up to the sky”.

- Level 1 (score: 1): the conflict is faced but tentatively, with a common ground based on features midway between physical and symbolic: Ex.: “Intelligence and skyscrapers are high and have many floors”; “It (“intelligence”) gets built gradually”; “It (“intelligence”) is something which goes higher and higher”.

- Level 2 (score: 2): the conflict is explicitly acknowledged and the structure of the metaphor is justified; both psychological characteristics of the tenor and physical characteristics of the vehicle are mentioned but on a clearly distinct basis. “It may mean that there are different levels of intelligence in people, as there are floors in skyscrapers”.

- Level 3 (score: 3): This is a refinement of the previous level, based on a deeper and more exhaustive analysis of the psychological characteristics of the target “intelligence”, in relation to the base “skyscraper”. Ex.: “You grow up in intelligence as you go upstairs, floor after floor, in a skyscraper. You can look at things from an upper level and understand them better, as in skyscrapers you discover things and they look small.” Maximum total score = 36. The MCT has high reliability, as measured by Cronbach’s alpha (0.70) and high interrater agreement, as measured by Cohen’s K (0.75 for the 9-year-olds and 0.81 for the 13-year-olds).

(2) A subtest, Idiom Comprehension (IC, henceforth), from a comprehensive battery to assess language competencies in children aged 4 to 12 (*Batteria per la Valutazione del Linguaggio* 4–12; Eng: *Language Assessment Battery*, LAB, henceforth) [51]. IC assesses the comprehension of 10 idiomatic sentences through multiple choice. Ex: “To lose oneself in a glass of water”. For each idiomatic sentence, three alternatives are given (a, b, c). In this case, (a) to be unable to face a small difficulty (correct); (b) to be unable to swim (literal interpretation); (c) to have a very big glass of water (metonymical interpretation). Each correct answer is assigned a point and the maximum total score is 10. IC has high reliability, as measured by Cronbach’s alpha (0.777) and test-retest correlation (0.932).

(3) A subtest, Iconic Metaphors (IM, henceforth) of a battery for children aged 5 to 14, the Abilità Pragmatiche del Linguaggio-Medea (It: APL-Medea; Eng: Pragmatic Language Abilities) [52]. Actually, this subtest does not assess the capability to understand metaphors but idioms and is composed of 4 items. The child is requested to choose an image that matches the meaning of expressions read by the examiner among four alternatives. Ex: “He always has his head in the clouds”. Scoring: 0 = incorrect answer; 2 = correct answer. Maximum total score = 8. The whole APL Medea battery has high reliability (Cronbach’s alpha = 0.922), and interrater agreement in the IM = 1.

(4) The NEPSY II Instructions Comprehension test (Instr. Comp., henceforth) [53,54], which assesses the ability to receive, process, and execute oral instructions of growing syntactic complexity. For each item, the child is requested to point to the appropriate stimuli in response to oral instructions. Ex (item 15): “Point to the blue cross and the red cross”. (Item 23): “Point to a shape that is between two crosses and above a circle”. Scoring: 0 = incorrect answer; 1 = correct answer. Maximum total score = 33.

We are aware that these tests do not cover the whole range of figurative language competencies but, to our knowledge, they were the only validated instruments for Italian pediatric population available when this study was conducted. In addition, as both the children with ASD and children with KS were recruited and assessed in a clinical context, the outcomes were part of the monitoring process. All the tests were administered by two psychologists especially trained for this type of assessment.

## 3. Results

The performance of the three groups of participants in each linguistic task of our study is reported in Table 1, in which the mean raw scores and standard deviations of each group are shown. It is clear that children in the ASD and KS groups had a lower performance than the TD controls in each linguistic task. The highest differences occurred in the MCT test, in which performance is examined on the basis of explicitness and level of abstraction in the explanations.

It can be observed that the standard deviation for the MCT test is very high in the ASD group. Actually, this high variability is generated by one participant with an outlier score of 24. In order to reduce the impact of this univariate outlier in our analysis, we followed Tabachnick and Fidell’s [55] suggestions and replaced the outlying score with the next most extreme in the score distribution of the ASD and KS groups (14, very close to the mean of TD children). Afterwards, the new values for MCT mean and standard deviation of the ASD group were 6.6 and 3.7, respectively.

To explore whether the performance of the three subgroups of children showed statistically significant differences we used a discriminant analysis [55].

The Wilks’s Lambda test in a discriminant analysis considers the overall relationship between the combination of predictors and the groups identifying the unexplained variance. In our analysis, the Wilks’s Lambda value was 0.347, which corresponds to a significant *F* value (*F*_(8, 48)_ = 4.19; *p* < 0.001). Thus, the combination of the four linguistic measures used in our study accounted for a significant 65.3% of the between-group variance (1-Lambda*100).

The second question that can be explored by means of a discriminant analysis is whether the combination of variables allowed to predict the participants’ group membership. The classification of participants, where actual group membership is compared to predicted group membership, showed that correct classifications were 80% for the TD group, 80% for the ASD group and 70% for the KS group. Thus, the combination of our linguistic measures was good at predicting membership to any of the three groups.

**Figure 1:**
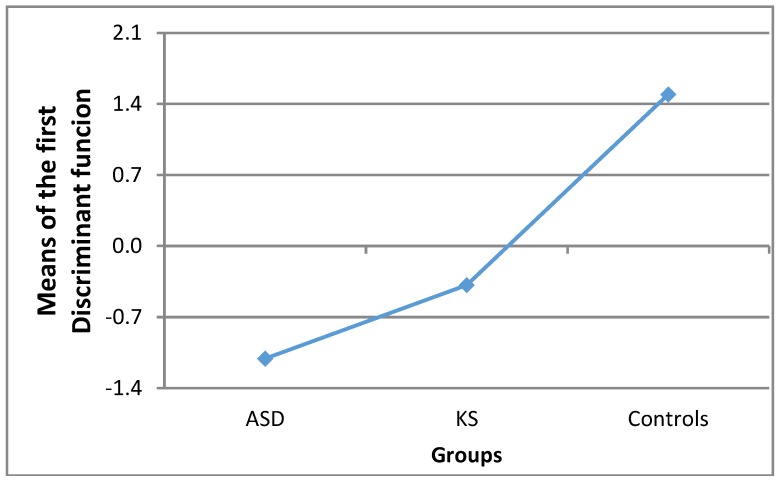
Means of the first discriminant function in the three groups of participants.

The third question explored by a discriminant analysis is which group was differentiated from the others in a statistically significant way. As there were three groups in our study, the analysis produced two discriminant functions but only the first turned out to be statistically significant (Chi-square = 26.99; *df* = 8; *p* < 0.001). Such function, as shown in Figure 1, maximally separated the TD group from the others. F-tests showed that the squared Mahalanobis distances between the TD group and both the ASD and the KS groups were significant (respectively: *F*_(4, 24)_ = 7.62; *p* < 0.001 and *F*_(4, 24)_ = 4.64; *p* < 0.01) whereas the distance between the ASD and the KS groups was not significant.

The fourth question faced by our analysis is whether the linguistic measures contributed with a similar weight to the discriminant function. The structure (loading) matrix of correlations between the predictors and the first discriminant function (see Table 2) showed that the best predictor for distinguishing between the TD group and the other groups was MCT, i.e., the test that assesses the capability to explain metaphors. In this case, the correlation with the discriminant function is much higher than for IC (Idiom Comprehension) and IM (Iconic Metaphors), based on the multiple- choice task.

In sum, the combination of linguistic abilities explored in our study differentiates in a statistically significant way TD participants from both ASD and KS participants. The ability to highlight the metaphoric relationship between tenor (e.g., intelligence) and vehicle (e.g., skyscraper) on explicit grounds is the best predictor of membership to a TD group versus a clinical group (either ASD or KS). In turn, this type of ability represents a form of reasoning on metaphors.

To clarify whether the low ability of reasoning with metaphors in children with ASD or KS was due to specific factors or to a general cognitive difficulty, we selected from our clinical sample only those participants whose total IQ was higher than 85. Mean raw scores and standard deviations in each linguistic task in the two clinical groups are reported in Table 3.

It clearly emerges that children with ASD or with KS had a lower performance than TD controls only in Instr.Compr. and in the MCT.

In this analysis, the Wilks’s Lambda value was 0.467, which corresponds to a significant F value (*F*_(8, 16)_ = 4.57; *p* < 0.01). Thus, the combination of the four linguistic measures used in our study accounted for a significant 53.3% of the between-group variance (1-Lambda*100). The classification of participants showed that correct classifications were 90% for the TD group, and 90% for the A + KS group. Thus, the combination of our linguistic measures was very good at predicting membership to both groups. In a significant discriminant analysis with two groups, only one (significant) function is produced and distances between groups are obviously significant.

The structure (loading) matrix of correlations between the predictors and the discriminant function (see Table 4) suggest that performances at Instr. Compr. remain an indicator of atypical development in the ASD and in the KS group of children, despite their IQ within normal range. However, the best predictor for distinguishing between the TD group and the two clinical populations was MCT. The correlation with the discriminant function was more than seven times stronger for MCT than for IM and IC.

## 4. Discussion

In the present study we wished to explore metaphor and idiom competencies in two clinical populations, namely children with ASD and children with KS. We approached the issue with different instruments, two multiple-choice tasks to assess idiom comprehension through iconic and verbal alternatives (IM and IC) and a metaphor explanation task (MCT). We are aware that the competencies these tasks assess are different in nature. In agreement with Winner [29], we believe multiple-choice tasks essentially assess *recognition* of a given form of figurative language within a range of pre-coded alternatives, conceived by adults, which might not reflect children’s spontaneous thoughts. On the other hand, *explanations* go beyond recognition and involve rephrasing capabilities, and these, in turn, require semantic and lexical competencies in addition to language planning abilities. We also agree with Winner’s statement [29] that a suitable explanation unequivocally reflects full comprehension. On these methodological grounds, we compared three groups of children (age range: 9–12), one with ASD, one with KS, and a TD group. The objective was to delineate the specific profiles of each of the two clinical groups concerning the different competencies, and, at the same time, to assess these groups against the TD controls’ profiles.

While idioms in IC’s and IM’s items were conventionalized forms, metaphors in MCT’s items were all novel, physical-psychological metaphors. In addition, we checked basic sentence comprehension through the Instructions Comprehension Test of the NEPSY II [53,54].

We hypothesized, in line with Kasirer and Mashal’s study [28], that we would not find differences between children with ASD and TD controls in multiple-choice tasks assessing idioms. We expected, instead, that differences would emerge in the task assessing metaphor explanation, where the child must explain on his/her own words a complex type of metaphors, namely novel, physical-psychological metaphors.

We performed a discriminant analysis that showed that idiom comprehension had a very small weight in distinguishing children with ASD from TD controls, whereas metaphor explanation did distinguish them even when children with ASD were selected for having an IQ higher than 85.

Thus, the outcomes of our study, in line with the main conclusions of Kalandadze et al. ii’s meta-analysis [27], suggest that figurative language comprehension is not a “core deficit” in individuals with ASD. Only when the task requires to explicitly construct and explain a semantic mapping between a concrete vehicle (e.g., skyscraper) and an abstract tenor (e.g., intelligence) the performance of children with ASD remarkably deviates from the typical population. This difficulty might be due to an abstractness factor. As argued by Jamrozik and colleagues [56] abstract concepts involve relations rather than collections of intrinsic properties. We may easily understand the characteristics that define “skyscrapers” and “intelligence” separately, but if we must project the characteristics of the former onto the meaning of the latter, we need to construe new relations. Such relational thinking might be particularly difficult for children with ASD due to their tendency towards local rather than global information processing, as pointed out by Happé and Frith [57]. Based on a recent study by Bambini et al. [58], it stil remains to clarify the role of ToM in typical development, as the relevance of this factor seems confirmed with respect to mental metaphors (psychological). The greater difficulty with psychological metaphors, especially in children with ASD, also emerges in a study conducted by Melogno and colleagues [59], where these children were treated in order to improve their abilities to process both sensory and psychological metaphors. Although an overall significant improvement was observed, this was far more evident in the sensory metaphors than in the psychological ones. Once more, the greater abstractness of these metaphors affected the performance [24].

Our sample of children with ASD also showed some difficulty in the Instructions comprehension task, that assesses basic syntactic competencies. This task proved to be the second variable contributing to differentiate children with ASD from the TD controls. This finding appeared even more evidently when the participants with ASD were selected for having an IQ higher than 85, and was also highlighted by a high correlation with the discriminant function. Thus, our sample of children with ASD had a weakness in sentence comprehension, and this weakness probably also affected tasks in which language had to be processed in depth, such as MCT.

Another aim of our study was to compare the two clinical populations to one another, the sample with ASD and the sample with KS. The outcomes of our study showed that all the aspects of the linguistic profile of children with KS were close to those of children with ASD, even when they had an IQ higher than 85. Thus, for children with KS, similarly to what was found in their peers with ASD, difficulties basically emerged in the explanation task (MCT), and these can presumably be linked to marked difficulties in expressive language, as pointed out by Leggett et al. [34].

We aware that the results of our study can only be considered as preliminary, either with respect to the respective profiles of two clinical populations, and with respect to the relationships between these profiles. This cautious position is due to the following limitations. The first is the reduced size of the sample. The second is the lack of IQ in the control group due to the reasons already pointed out. The third regards a part of the assessment, namely Instructions comprehension, where semantic and syntactic competencies could have been explored also at the productive level. The fourth limitation is that we only considered quantitative outcomes, based on the type of statistical processing we used to highlight major differences.

At the same time, we believe this study has also some elements of originality. First, it investigated a clinical population scarcely explored from the point of view of figurative language competencies, namely children with KS [35,38], and in relation with another clinical population, that of children with ASD [27].

Secondly, the age range, which we circumscribed to the interval from 9 to 12, targets a significant period in the development of figurative language in children without neurodevelopmental disorder [29].

Thirdly, we assessed two different types of competencies—recognition (idioms) and explanation (metaphors)—generally confused under the overall term “comprehension” [29]. These could be considered as two subcomponents of the processing mechanisms in the figurative language macroarea. On speculative grounds, we might view these subcomponents as an expression of microabilities. In this vein, we may cite a recent research line on children with ASD that highlighted anomalies in basic non social mechanisms, such as multisensory integration but also in language development [60,61,62]. These anomalies suggest that they could also be generated by microabilities. A further promising comparison between individuals with ASD and another clinical population could be between individuals with ASD and individuals with the Cerebellar Cognitive Affective Syndrome, described in the recent review article by Casartelli and colleagues [63].

As a fourth point, we will mention the relevance of the above distinction between recognition and explanation for clinical assessment. For example, when assessing children with KS or children with ASD, using only multiple-choice tasks might overestimate their performance. We will mention an example drawn from our study, for the Italian idiom “Prendere un granchio” (literally: “To take a crab”, meaning “to make a gross error”) where the possible answers were:

Answer n.1: to make a mistake

Answer n 2: to take a crab with one’s hands

Answer n 3: to go to the seaside.

A child with ASD said: “I withdraw the word ‘crab’ from the second answer, the word ‘seaside’ from the third answer because ‘crab’ and ‘seaside’ have something in common with the expression “To take a crab”, then, the right answer is the first one”. Apparently, then, the child was aware of the non literal meaning of this expression and also that he did not know it. In this situation, he opted for a default strategy that led him to the correct answer. While this may seem an adaptive metacognitive strategy it does not warrant that the child really grasped the meaning in depth. These cognitive pathways are precisely what should be investigated in future research if we wish to illuminate reasoning about figurative language in development.

## 5. Conclusions

In this study we explored two different aspects of the multidimensional figurative language domain in two clinical populations, children with autistic spectrum disorder (ASD) and children with Klinefelter syndrome (KS), compared to typically developing (TD) children. In particular, we studied the capability to recognize the meanings of idioms in multiple-choice tasks and the capability to explain novel metaphors in a task that explicitly requested to reason on metaphors. The outcomes of this study, although preliminary, support the idea to distinguish different components in figurative language competency. On the one hand, this distinction could highlight strenghts and weaknesses in the abovementioned clinical populations, and, on the other, it could also illuminate the complex computational mechanisms underlying performances that might superficially appear as adequate. 

## Figures and Tables

**Table 1:** Mean raw score and standard deviation in Instr. Compr., Idiom Comprehension (IC), Iconic Metaphors (IM), and Metaphor Comprehension Test (MCT), ASD: autistic spectrum disorder; KS: Klinefelter syndrome; TD: typically developing.

Groups	Instr. Comp.	IC	IM	MCT
ASD	23.2 (2.2)	7.3 (2.6)	5 (2.5)	7.6 (6.3)
KS	24.2 (2.2)	6.5 (2.6)	3.8 (1.5)	7.7 (3.6)
TD Controls	26.9 (3.4)	8.5 (1.1)	6 (1)	15.7 (4.6)

**Table 2:** Correlation matrix between predictors and the first discriminant function.

Predictors	Correlations with the Discriminant Function
MCT	0.911
Instr. Comp.	0.477
IM	0.304
IC	0.262

**Table 3:** Mean raw scores and standard deviations in Instr. Compr., IC, IM and MCT.

Groups	Instr. Comp.	IC	IM	MCT
ASD or KS (*N* = 11)	24.5 (3.4)	8.2 (1.3)	5.6 (1.5)	9.5 (4.7)
Controls (*N* = 10)	26.9 (3.4)	8.5 (1.1)	6 (1)	15.7 (4.6)

**Table 4:** Correlation Matrix between predictors and the discriminant function (ASD group or KS group with total IQ higher than 85 versus TD Controls).

Predictors	Correlations with the Discriminant Function
MCT	0.832
Instr. Comp.	0.355
IM	0.117
IC	0.114

## Appendix A

**Table A1:** Parents’ and teachers’ informations about the TD control group.

Parents/Teachers Children	1	2	3	4	5	6	7	8	9	10
Developmental disorders within the family	−	−	−	−	−	−	−	−	−	−
Previous developmental disorder (ex. language disorder)	−	−	−	−	−	−	−	−	−	−
Previous or current behavioral difficulties	−	−	−	−	−	−	−	−	−	−
Difficulties in the initial Stages of school learning	−	−	−	−	−	−	−	−	−	−
Adequate reading (word decoding)	+	+	+	+	+	+	+	+	+	+
Adequate reading (comprehension)	+	+	+	+	+	+	+	+	+	+
Adequate oral production (conversation and narrative)	+	+	+	+	+	+	+	+	+	+

Legend: the sign − stands for absence and the sign + stands for presence.
